# A low-cost triangular water-filled stand-off pad to improve needle visibility in ultrasound-guided procedures

**DOI:** 10.1186/s42155-026-00692-w

**Published:** 2026-04-24

**Authors:** Yuji Kumakura, Takayuki Asao, Makoto Sakai, Kengo Kuriyama, Takuhisa Okada, Takuya Shiraishi, Akiharu Kimura, Akihiko Sano, Ken Shirabe, Hiroshi Saeki

**Affiliations:** 1 Department of General Surgical Science, Graduate School of Medicine, Gunma University, 3-39-15 Showa-machi, Maebashi, Gunma Japan; 2 Innovative Research & Liaison Organization, Shinshu University, Matsumoto, Japan

To the Editor,

It is well established that needle visibility during ultrasound-guided procedures improves when the beam–needle angle is shallow, as specular reflections are directed back toward the transducer [1]. However, in actual clinical practice, adjustment of the insertion angle is often limited by the location of the target, surrounding anatomy, or procedural constraints, making optimal beam–needle alignment difficult to achieve.

To address this limitation, the use of stand-off pads or gel pads has been reported as a method to improve visualization by increasing the distance between the probe and the target [2]. However, commercially available gel stand-off pads are single-use, dedicated devices and are not routinely stocked in many ultrasound suites, which may limit their widespread use, particularly in time-sensitive or bedside procedures.

Signal-to-noise ratio (SNR) has been reported as a quantitative method for evaluating ultrasound needle visibility and echogenicity [3]. This objective metric enables assessment beyond subjective visual impression and allows comparison of different techniques for improving needle visualization.

In this context, we developed a simple and inexpensive stand-off pad using a standard plastic bag filled with water. Unlike conventional approaches in which a sterile probe cover and gel create a flat interface between the probe and the surface, our design utilizes the corner of the plastic bag to form a triangular stand-off structure (Fig. 1A, B). In this configuration, the ultrasound probe is placed in direct contact with the water, which serves as the acoustic coupling medium. This configuration locally elevates and tilts the ultrasound probe, thereby bringing the effective beam–needle angle closer to horizontal and improving needle visibility.

**Fig. 1:**
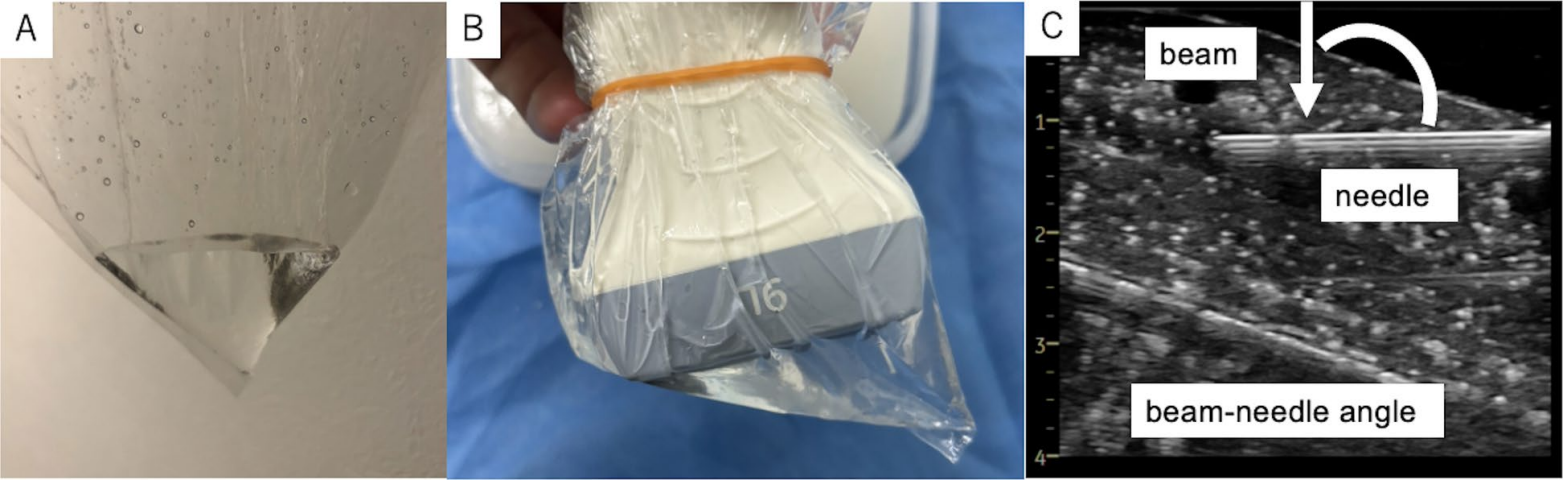
Construction and representative use of the triangular water-filled stand-off pad. **A**, **B** A standard polyethylene bag is used, with approximately 30 mL of water pooled and secured into one corner using a rubber band, forming a compact triangular stand-off pad. **C** With the stand-off pad in place, the effective beam–needle angle becomes closer to horizontal, resulting in improved needle visibility. The beam–needle angle is schematically indicated, and the skin entry point becomes clearly identifiable

With the use of this triangular stand-off pad, the needle shaft was clearly identifiable under ultrasound guidance. Importantly, the skin entry point was also more clearly visualized, facilitating confirmation of the puncture site (Fig. 1C). These improvements were achieved without altering the needle insertion path or requiring additional equipment. All experiments were conducted using a vascular ultrasound training phantom (UGP-GEL, Alpha Bio, Japan).

The regions of interest used for SNR calculation are illustrated in Fig. 2A. Quantitative analysis demonstrated a significant improvement in needle visibility when the stand-off pad was used (Fig. 2B). The SNR increased from 6.08 ± 0.49 without the pad to 11.22 ± 1.97 with the pad (mean ± SD, *n* = 10), representing nearly a twofold enhancement under identical imaging parameters (*p* < 0.001). Furthermore, to evaluate compatibility with sterile technique, imaging was performed using both single-layer and double-layer sterile probe coverings. The average SNR was 11.22 ± 1.97 with a single-layer cover and 12.52 ± 2.75 with a double-layer cover, with no significant difference between the two conditions (*p* > 0.1, Fig. 2C), indicating that needle visibility was not compromised by additional sterile barriers.

**Fig. 2:**
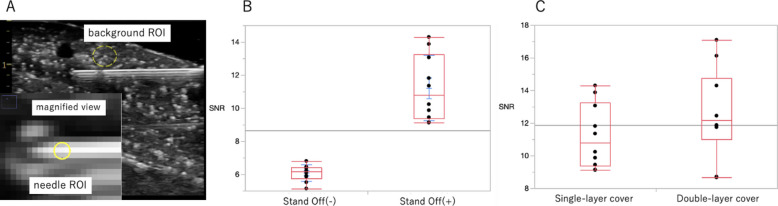
Quantitative analysis of needle visibility using signal-to-noise ratio (SNR). **A** Representative ultrasound image illustrating the placement of regions of interest (ROIs) used for SNR calculation. The background ROI was placed in a homogeneous phantom region adjacent to the needle while avoiding vessel-like echo-free spaces. The needle ROI was defined on the echogenic portion of the needle tip in a magnified view. **B** The triangular water-filled stand-off pad significantly improves needle visibility compared with no-pad conditions. **C** Comparison of SNR between single- and double-layer sterile covers shows no significant difference, indicating that sterile covering does not impair needle visibility

Because this triangular stand-off pad can be constructed easily from inexpensive and readily available materials, it is well suited for routine use in ultrasound-guided vascular access, particularly in anatomically constrained situations where optimization of the beam–needle angle is difficult. In addition, although the present study focuses on vascular applications, the same geometric principle may be applicable to other ultrasound-guided needle procedures, such as regional anesthesia, where needle visibility is similarly angle dependent. One potential limitation is that the tilted probe configuration may slightly shift the apparent skin entry point for inexperienced operators. Preprocedural skin marking may help maintain puncture accuracy.

## Data Availability

Not applicable.
